# Supplementary figures and images for: Generation of a Transgenic Zebrafish Line for In Vivo Assessment of Hepatic Apoptosis
Source: Pharmaceuticals (Basel). 2021 Oct 31;14(11):1117. doi: 10.3390/ph14111117 (PMC8618266; doi:10.3390/ph14111117)

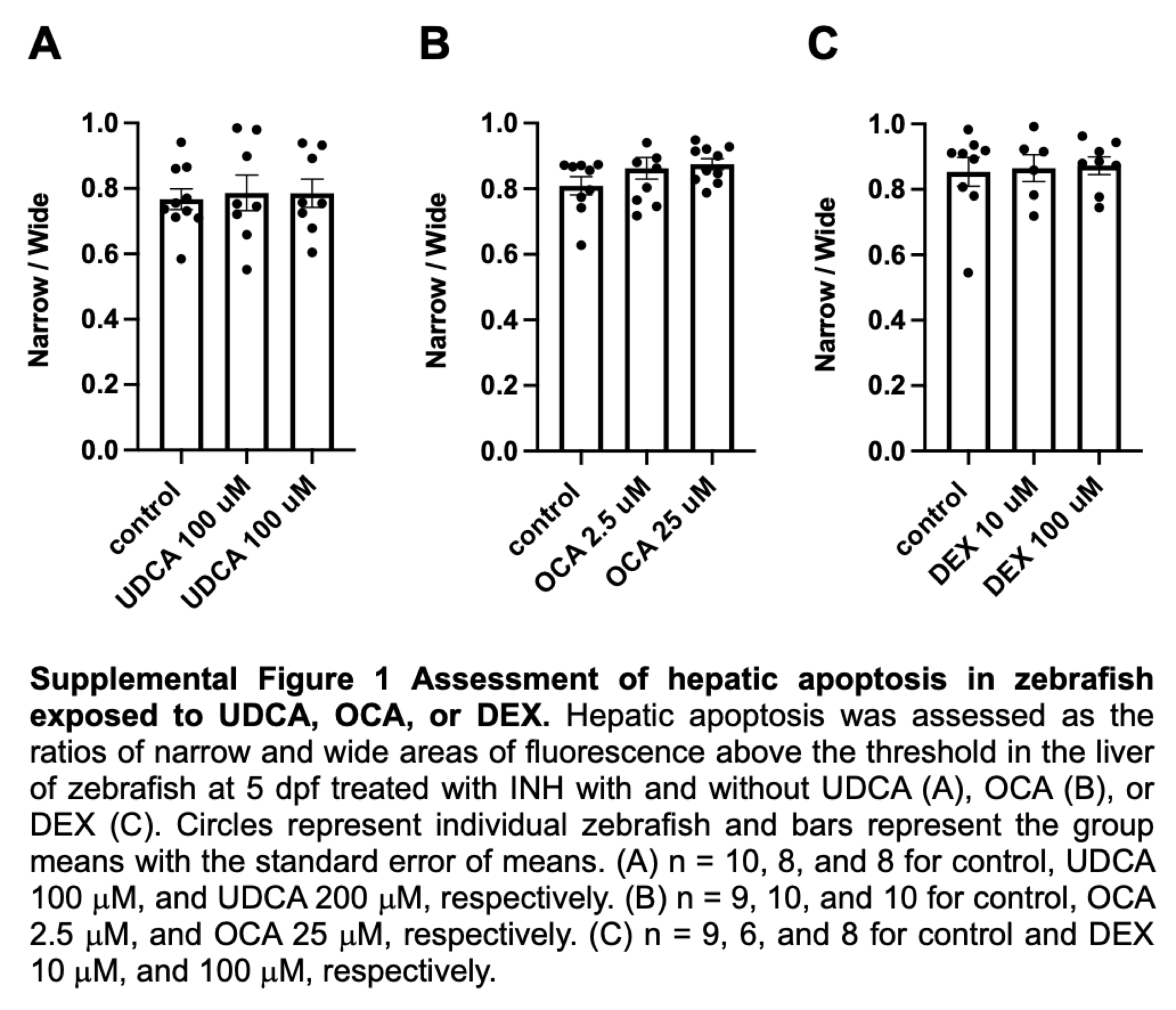

Supplement: Supplementary file 1 [file pharmaceuticals-14-01117-s001.zip › pharmaceuticals-1449145-supplementary.png]
